# Case of an Extra Uterine Fœtus

**Published:** 1784

**Authors:** 


IV. *Case of an extra Uterine Fœtus. By Mr.* Cammel, *Surgeon at Bungay, in Suffolk. Communicated to Dr.* Simmons *by Dr.* Denman.

MS. a healthy woman, about thirty-five years of age, had been married several years, but had never reason to suspect herself pregnant. In the beginning of May, 1782, she had a profuse menstrual discharge, which continued for fourteen successive weeks, but from the time of its cessation, she did not menstruate for six months.

In the month of October following, I saw her for the first time, and was informed that she

ſhe had been frequently liable to a painful ſuppreſſion of urine, for the relief of which it had often been neceſſary to introduce the catheter. On examination, I could readily diſtinguiſh a tumour of a conſiderable ſize, between the *vagina* and *rectum*, and the *os uteri* turned upwards and forwards towards the pubes. I immediately concluded ſhe was with child, and that the *uterus* was retroverted. It was agreed that nothing ſhould be done, except it ſhould be neceſſary to draw off the urine with the catheter, or to obviate any troubleſome ſymptom which might ariſe. On the following day, I returned to London, and did not ſee the patient again before June 1783, when ſhe told me the ſuppreſſion of urine had not returned ſince the time of my firſt viſiting her, but that ſhe had continually increaſed in ſize; and ſhe was now as big as women uſually are in the eighth month of pregnancy. I was permitted to make another examination, and found the tumour between the *vagina* and *rectum*. The *os uteri* was preſſed low into the *vagina*, but the *cervix* was of the length and in the ſtate it is in when women are not with child. She alſo informed me that ſhe had menſtruated regularly ſince the beginning of February, and as I perceived the *abdomen* to be more more diſtended on the right than on the left ſide, I laid aſide my opinion of her being pregnant, and ſuſpected that the enlargement and diſeaſe of the right *ovarium* was the cauſe of the diſtenſion, and by mechanical preſſure, of the other ſymptoms. During the time of this gradual enlargement, ſhe had frequent and ſometimes violent pains in the *abdomen*; but as theſe changed their ſituation, ſhe could not fix upon any one part as particularly affected.

In this ſtate, or without much alteration, ſhe continued till April 1784; then the pains became more violent, attended with a hectic fever, and very frequent ſtools, in which there was always a quantity of ſanious and ſometimes putrid matter, by which the ſize of the *abdomen* was ſomewhat leſſened. I attributed theſe ſymptoms to the progreſs of the diſeaſe in the *ovarium*, which I ſuppoſed to have become cancerous; and, together with other medicines, I gave her the bark, and opiates whenever the violence of the pain required them. About the latter end of May, after complaining of a pricking ſenſation in the *rectum*, ſhe voided a ſmall bone, by ſtool. When I examined the ſtate of that part, I diſcovered, very far within the *rectum*, an aperture through the inteſtine, leading leading to the right ſide of the *pelvis*, where the aperture was moſt obvious, and where I diſcovered many other ſmall bones, which I then ſuſpected muſt have been thoſe of an extra-uterine *fœtus*. With a pair of forceps uſed in lithotomy, theſe were extracted; but the bones of the head being too large, I broke them in pieces, before I attempted to extract them. In two hours, I had removed all the bones, except one of the parietal, which was ſo firmly embraced, as to render the extraction very difficult. As ſhe was very much fatigued with what was already done, I waited two days before I took away this bone. Nothing farther remarkable happened after the operation, except that a large quantity of mucous water was diſcharged with the ſtools; but this in a ſhort time decreaſed, and then ceaſed entirely.—Within a ſhort time, the diarrhœa and fever abated, and in about ſix weeks ſhe was able to return to her uſual employment, and is now perfectly well.

